# Prevalence of Behavioural and Psychological Symptoms of Dementia in Individuals with Learning Disabilities

**DOI:** 10.3390/diagnostics5040564

**Published:** 2015-12-02

**Authors:** Rajal Devshi, Sarah Shaw, Jordan Elliott-King, Eef Hogervorst, Avinash Hiremath, Latha Velayudhan, Satheesh Kumar, Sarah Baillon, Stephan Bandelow

**Affiliations:** 1 Loughborough University, School of Sport, Exercise and Health Sciences, Loughborough, Leicestershire LE11 3TU, UK; E-Mails: s.shaw-10@student.lboro.ac.uk (S.S.); j.elliott-king@lboro.ac.uk (J.E.-K.); e.hogervorst@lboro.ac.uk (E.H.); s.bandelow@lboro.ac.uk (S.B.); 2 Learning Disabilities Services, Leicestershire Partnership NHS Trust, Mansion House, Leicester Frith Hospital site, Groby Road, Leicester LE3 9QF, UK; E-Mails: avinash.hiremath@leicspart.nhs.uk (A.H.); satheesh.kumar@leicspart.nhs.uk (S.K.); 3 Mental Health Services for Older People, Leicestershire Partnership NHS Trust, The Evington Centre, Gwendolen Road, Leicester LE5 4QG, UK; E-Mail: lv24@leicester.ac.uk; 4 Department of Health Sciences, University of Leicester, Leicester General Hospital, Gwendolen Road, Leicester LE5 4PW, UK; E-Mail: sfb5@leicester.ac.uk

**Keywords:** BPSD, learning disabilities, dementia, neuropsychiatric inventory

## Abstract

A review of 23 studies investigating the prevalence of Behavioural and psychological symptoms of dementia (BPSD) in the general and learning disability population and measures used to assess BPSD was carried out. BPSD are non-cognitive symptoms, which constitute as a major component of dementia regardless of its subtype Research has indicated that there is a high prevalence of BPSD in the general dementia population. There are limited studies, which investigate the prevalence of BPSD within individuals who have learning disabilities and dementia. Findings suggest BPSDs are present within individuals with learning disabilities and dementia. Future research should use updated tools for investigating the prevalence of BPSD within individuals with learning disabilities and dementia.

## 1. Introduction

Dementia is a clinical syndrome, which is characterised by the deterioration of mental functioning. This includes the development of various cognitive and intellectual deficits, which can affect the ability to carry out everyday tasks [1]. There is no known cure for progressive dementias, however a variety of treatments are available to alleviate the associated symptoms [2]. Dementia is a degenerative condition, most prominent within older adults. An estimated one million people in the UK will have dementia by 2025 [3]. Dementia has a costly impact on the economy; currently costing the UK over £26 billion a year to cover health and social care costs [3,4].

Over time, the general life expectancy of individuals with learning disabilities has steadily increased [5] thus leading to dementia becoming a growing issue within this population. Learning disabilities is an umbrella term covering a range of difficulties which can interfere with the acquisition and use of reasoning, writing and reading skills, as well as other intellectual, social abilities [6]. Previous research has indicated that the prevalence of dementia is greater in people with learning disabilities as opposed to the general population [7,8]. Research has established that there is an increased risk of early onset dementia of the Alzheimer type in people who have Down’s syndrome [9,10,11]. Findings have suggested that one in three people with Down’s syndrome will develop dementia in their 50s, as well as over half of those who live to the age of 60 or over [12].

A widespread series of behavioural and psychological symptoms are common in dementia, some of which include apathy, aggression, delusions, psychosis, hallucinations anxiety, irritability, eating disturbances and sleep disorders [13]. Behavioural and psychological symptoms of dementia (BPSD) are non-cognitive symptoms, which constitute as a major component of dementia regardless of its subtype [3]. BPSD often occur in clusters such as hyperactivity, including agitation and aggression, affective symptoms including depression and anxiety, and psychosis, including both delusions and hallucinations [13]. The prevalence of specific symptoms vary between study to study. In general, hallucinations have been reported in 15%–49% of patients with dementia, delusions have been found in 10%–73% of dementia patients. Affective symptoms such as depression are less common; however, this could be due to the difficulty in differentiating between symptoms of depression and dementia as they can both co-occur. Incidences of behavioural disturbances such as agitation and aggression have been found to be highly prevalent within people who have dementia, with studies reporting a mean prevalence of 46.2% [14].

Behavioural and psychological symptoms cause considerable distress to both the patient and carer, and could potentially reduce their quality of life [15]. Furthermore, research has indicated that BPSD can be significant predictors of institutionalisation [16,17]. The nature of BPSD is not fully understood; therefore the treatment options are minimal. Pharmacological treatments alleviate specific symptoms, examples of which include the use anti-dementia drugs such as Memantine and antipsychotics such as Olanzapine and Risperidone [18]. The use and efficacy of non-pharmacological interventions, such as Snoezelen (multi-sensory stimulation), have also been investigated throughout research [19].

The purpose of this literature review is to provide a critical evaluation the methodology of research exploring the prevalence of BPSD within individuals who have to learning disabilities and dementia.

## 2. Literature Search

There is no global definition of BPSD. The behavioural and psychological symptoms of dementia were previous recognised as neuropsychiatric, maladaptive or non-cognitive behavioural disturbances [20]. For the purposes of this literature review the following definition of BPSD was used “Signs and symptoms of disturbed perception, thought content, mood, or behaviour that frequently occur in patients with dementia” [21]. The literature search was carried out using the search engines such as Google scholar and databases, such as PubMed, Wiley, Science Direct, Cambridge journals, and Psych Articles. Combinations of key search terms and phrases used are shown below.

Terms:

- BPSD
- Learning disabilities
- Dementia
- BPSD Prevalence
- Neuropsychiatric inventory
- Psychiatric symptoms
- Neuropsychiatric symptoms
- Non cognitive symptoms
- Mood changes
- Maladaptive behaviours
- Behavioural disturbances
- Psychological changes

### Inclusion and Exclusion Criteria

Inclusion and exclusion criteria aid in selecting relevant studies. Research articles that focused on the non-cognitive symptoms of dementia in learning disabilities were included. Studies that did not focus on the aims of this literature review, such as articles based on treatment of BPSD, were excluded for the purposes of this literature review. Furthermore, when searching for BPSD in the general population, articles, which had population samples under 60, were excluded, as prevalence rates would be difficult to compare to other studies with larger sample sizes.

## 3. Prevalence of BPSD in the General Population

Behavioural and psychological symptoms of dementia (BPSD) effect up to 90% of those diagnosed with dementia at any given point in the duration of their illness [3]. There have been various studies that have investigated the prevalence of BPSD within the general population (see Table 1). Savva *et al.* [22] conducted a longitudinal population based study over a period of 10 years. BPSD prevalence was assessed in people with and without dementia. The results suggested that each BPSD excluding sleep disturbance was significantly higher in people with dementia compared to those without dementia. Haupt, Kurz and Jenner [23], reported similar findings within their two year study, stating that all participants experienced at least one BPSD within the duration of the study. There are major flaws, which weaken the overall findings of longitudinal studies. Sample sizes are difficult to maintain in longitudinal studies. Savva *et al.* [22], reported that a total of 587 participants who had dementia and 2050 that did not have dementia were used for the baseline assessment. However, only 244 participants were reassessed after 22 months (42%).

**Table 1:** Studies investigating behavioural and psychological symptoms of dementia (BPSD) in the general population.

Author, Year, Country	Aim	Measure Used to Assess Behavioural and Psychological Symptoms of Dementia (BPSD)	Participants (*n*)	Main Findings
Savva *et al.* [22], UK	To describe the prevalence, correlates and course of BPSD in the population of England and Wales	Cambridge Mental Disorders of the Elderly Examination CAMDEX Geriatric Mental State (GMS) interview	587	Prevalence in dementia participants was significantly higher than non-dementia group apathy was highest (50.3%) followed by irritability (28.8)
Chiu, Chen, Yip, Hau and Tang [24], Taiwan	Prevalence of BPSD in patients with four major types of dementia	Behavioural Pathology in Alzheimer’s Disease Rating Scale (BEHAVE-AD)	137	BPSD were found in 92.0% of the patients
Margallo-Lana *et al.*, [25], UK	To determine the prevalence of BPSD in care environments, their relationship with severity of dementia and the pattern of psychotropic medication	Neuropsychiatric inventory (NPI)	231	79% of participants had clinically significant BPSD
Ballard *et al.*, [26], UK	Examining the prevalence, incidence, and outcome of the 3 main BPSD (agitation, depression, and psychosis) in care facilities	Neuropsychiatric inventory (NPI)	136	Prevalence of BPSD was 76% at baseline and 82% at follow-up
Brodaty *et al.*, [27], Australia	To investigate the prevalence of BPSD in nursing home residents using the BEHAVE-AD	BEHAVE-AD (Behavioural Pathology in Alzheimer’s Disease)	647	Over 90% of participants rated positive for BPSD
Habio *et al.*, [28], China	Study of the prevalence of BPSD in population based community-living persons with dementia	NPI (Neuropsychiatric inventory)	1271	50.1% had at least one BPSD
Haupt, Kurz and Janner [23], Germany	Examine the longitudinal occurrence and persistence of BPSD in Alzheimer’s disease	Behavioural Abnormalities in AD Rating scale (BEHAVE-AD)	60	100% of participants experienced BPSD over 2 year period

The overall reliability of the findings would be questionable as less than half the participants provided a second interview, thus comparisons made with overall baseline measure would not be completely valid unless their data was excluded. Furthermore, the participant groups were far from balanced, the prevalence of BPSD was compared between 587 participants with dementia and 2050 without dementia. However, the general results from the study-mirrored findings from other longitudinal studies (Chache Country study and Nakayama study) so in this case the imbalance was not a significant issue.

The use of smaller sample sizes could increase the validity and reliability of longitudinal studies, Haupt, Kurz and Jenner [23] used a sample of 60 dementia participants to investigate the prevalence of BPSD, all of which were available for follow up BPSD assessments over a two-year period. A final issue with the use of longitudinal studies is the inability to record constant observations. Both Savva *et al.* [22] and Haupt, Kurz and Jenner [23] were not able to observe any short-term fluctuations or short episodes of symptoms.

BPSD prevalence rates of 79% and above were reported in the five of the eight studies [23,24,25,26,27,28]. This indicates a consistency of reliable results across all five studies; however, a variety of measures were used to assess BPSD. One measure used to assess BPSD is the behavioural pathology in Alzheimer’s disease (BEHAVE-AD). BEHAVE-AD is a well-known, valid measure of BPSD, which consists of a set of questions given to an informant during an interview with a clinician. Chiu, Chen, Yip, Hau and Tang [24] Brodaty *et al.* [27] and Haupt, Kurz and Janner [23] used BEHAVE-AD as the main form of BPSD assessment. Their results were found be highly consistent with each other, all reporting a BPSD prevalence at 90% or over (see Table 1). Therefore, indicating the use of BEHAVE-AD to assess BPSD as a valid measure. The remaining studies [25,26,28] (see Table 1) used the Neuropsychiatric inventory (NPI) also reported similar findings between each other.

In conclusion, research has indicated that there is a high prevalence of BPSD in the general dementia population. The studies used to investigate the prevalence of BPSD in the general population reported consistent findings. The use of different measures to assess BPSD did not affect the overall reliability of the results, as both the NPI and BEHAVE-AD are highly valid tools used to assess BPSD in multiple studies. The population samples used in the studies were of a significant size, thus further increasing the reliability and ecological validity of the general findings.

## 4. Prevalence of BPSD in the Learning Disability Population

There are limited studies that investigate the prevalence of BPSD within individuals who have learning disabilities and dementia. Due to the lack of research, it is difficult to provide a general consensus regarding the prevalence of BPSD within this specific population sample. During the initial literature search, “BPSD in learning disabilities and dementia” yielded no valid results in any database. However using a combination of other terms (see Section 2) eight appropriate studies were selected (see Table 2).

The studies do not investigate the prevalence of BPSD as a whole in the learning disability population. Each study focuses either on the behavioural or psychiatric symptoms of dementia. Aggressive behaviour is a common problematic behavioural symptom of dementia [29]. Duggan, Lewis and Morgan [30] and Cooper and Prasher [31] reported a high prevalence of aggressive behaviour within individuals with learning disabilities and dementia. Apathy and depression are both behavioural problems prevalent in dementia. Depression has been described in 0%–87% of dementia patients. Approximately 80% of Alzheimer’s disease patients are apathetic during the progression of their illness [32]. Depression and apathy have also been reported in studies examining participants with learning disability and dementia. Burt, Loveland and Lewis [33] suggested that depression and dementia were associated in people who have Down’s syndrome.

**Table 2:** Studies investigating behavioural and/or psychiatric symptoms within individuals with learning disabilities and dementia.

Author, Year, Country	Aim	Measure	Participants (*n*)	Main Findings
Duggan, Lewis and Morgan [30], UK	Investigating behavioural changes in individuals with learning disability and dementia	Past Behaviour Health Inventory (PBHI)	12	Reported aggression, sleep and eating disturbances in participants
Prasher and Filer [35], UK	Investigating behavioural disturbances in people with downs syndrome and dementia	Full access to article was unavailable	45	Reported findings of low mood, sleep disturbance and wandering in participants with dementia and downs syndrome
Cooper and Prasher [31], UK	Compared the occurrence of maladaptive behaviours in individuals with dementia with and without downs syndrome	Present Psychiatric State Learning Disability Scale (PPSD)	134	Reported high prevalence of aggressive behaviour (61.5%)
Cooper [7], UK	Determine the rate of psychiatric symptoms amongst the elderly with learning disabilities and dementia	Present Psychiatric State Learning Disability Scale (PPSD)	143	Psychiatric symptoms reported in 27.6% participants
Moss and Patel [37], UK	Psychiatric symptoms associated with dementia in older people with learning disability	Psychiatric Assessment Schedule for Adults with a Developmental Disability (PAS-ADD)	105	Participants with definite dementia had higher levels of sleep difficulty, hypersomnia, irritability, inefficient thought, loss of interest and anhedonia
Burt, Loveland and Lewis [33], USA	Examined the relation between dementia and depression in 61 adults with Down and 43 adults with mental retardation	Neuropsychological battery to assess declines in functioning	104	Results suggest that dementia and depression were associated in downs syndrome
Prasher [35], UK	Investigated age-specific prevalence, thyroid dysfunction and depressive symptomatology in adults with Down syndrome and dementia	Full access to article was unavailable	201	Depressive symptomatology of depressed mood, weight loss and reduced appetite were associated with dementia in adults with downs syndrome
Moss and Patel [36], UK	Investigated symptoms of physical and mental illness and levels of adaptive behaviour in adult with dementia and intellectual disabilities	Psychiatric Assessment Schedule for Adults with a Developmental Disability (PAS-ADD)	101	Reported sleep difficulty, hypersomnia and irritability as significantly severe in adults with dementia and intellectual disability

Prasher and Filer [34] provide further support for these results, reporting findings of low mood in 134 participants who had Down’s syndrome and dementia. Prasher [35] replicated findings in a larger study using 201 participants and described depressive symptomology in adults with Down’s syndrome and dementia.

Over half the studies (62.5%) selected (see Table 2) state a high prevalence of sleep disturbances within people with learning disabilities and dementia. Savva *et al.* [22] mirrored these results in patients with dementia without learning disabilities. Cooper [7] investigated psychiatric symptoms in adults with learning disabilities and dementia in a population study. The results suggested that 27.6% of participants suffered from psychiatric symptoms. Highly prevalent symptoms reported from the studies investigating behavioural and or psychiatric symptoms of dementia in adults with learning disabilities (see Table 2) have also been reported in the general dementia population (see Table 1). It is evident that behavioural and psychological symptoms are present in adults with dementia whether they have a learning disability or not.

A general issue with the studies investigating BPSD relates to the type of measure used for assessment. There is no gold-standard instrument to measure BPSD in the learning disability population. Dugan, Lewis and Morgan [30] used the past behaviour inventory (PBHI). PBHI is an out-dated self-report questionnaire; there are many updated behavioural measures for assessing BPSD, such as the neuropsychiatric inventory. The PBHI is limited to reporting behavioural issues and does not account for any psychological behaviour. Therefore, the PBHI would not be able to assess any of the psychological elements relating to BPSD, Furthermore, the PBHI questionnaire relies completely on self-report, patients displaying BPSD may not be able to distinguish between behavioural and psychological symptoms. Additionally, patients may also not be aware of presenting any symptoms. The measure is also not specified for patients with dementia. Therefore, it can be concluded that the PHBI is a not a valid measure for BPSD.

Additional tools used for assessing BPSD in the learning disability population include the Present Psychiatric State Learning disability Scale (PPSD) and Psychiatric Assessment Schedule for Adults with Developmental Disabilities (PAS-ADD). Cooper and Prasher [31] and Cooper [7] both used the PPSD when investigating maladaptive and psychiatric behaviours in adults with learning disabilities. Moss and Patel [36,37] used the updated version, PAS-ADD. Both measures are semi-structured interviews, specifically designed for the detection of mental disorders in people with intellectual disabilities. The PAS-ADD and the PPSD are not valid measures of assessing BPSD. As they focus solely on the psychiatric symptoms and states of participants with learning disabilities. Therefore, both the PAS-ADD and PPSD are unable to assess any of the behavioural aspects of BPSD. Detailed behavioural and psychological behaviours can be difficult to assess with these scales. Furthermore, neither scale is specified for detecting symptoms in patients with dementia. In general, the overall strength of the PAS-ADD is that it is suitable to use for the learning disability population. The questions are especially developed and adapted for the specific population. The PHBI however, is more of a general behavioural measure, which can be administered to anyone in the general population. An additional instrument used to assess BPSD is the BEHAVE-AD scale. BEHAVE-AD assess behavioural and psychological elements of Alzheimer disease, however due to the specific focus on symptoms associated with Alzheimer’s disease the scale may not be generalizable.

The PBHI, PAS-ADD and PPSD have various flaws in regards to the assessment of BPSD. None of the three scales mentioned assess both the behavioural and psychological symptoms associated with dementia. Thus, the scales would not be able to provide an accurate assessment of BPSD in the learning disability population.

To conclude, the measures used in the studies are difficult to validate, thus making methodological comparisons challenging. The most evident comparison is between Moss and Patel [36,37] both studies reported a high prevalence of sleep difficulty, hypersomnia and irritability with the use of PAS-ADD. All eight studies were conducted in the 1990s; BPSD was a relatively new and developing term during this period. This may explain the lack of research within the selected population and the use of out-dated measures. Future research within this area should use updated tools for investigating the prevalence of BPSD within individuals with learning disabilities and dementia.

## 5. Use of Neuropsychiatric Inventory (NPI)

The vast majority of recent studies of have used the Neuropsychiatric Inventory (NPI) to assess BPSD (see Table 3). The NPI was developed by Cummings *et al.* [38]. The NPI is administered by the clinician to the caregiver. The caregiver is usually a family member involved in the daily care of the patient. The NPI can be administered to a professional caregiver or other involved person as long as they have detailed knowledge of the patient’s behaviour. Multiple Studies have demonstrated the content and concurrent validity, as well as between-rater, test-retest, and internal consistency reliability of the NPI. The NPI is both a valid and reliable measure of BPSD. The NPI has the advantages of evaluating a wider range of psychopathology than existing instruments, whilst minimizing administration time [38]. There are several versions of the NPI, such as the NPI-Q, NPI-NH and the NPI-C. Some versions have also been translated into various languages.

Studies that have used the NPI to assess BPSD, have reported similar results. The prevalence of BPSD in the population has been estimated in various well known longitudinal studies (Nakayama and the Cache County study) Each of these studies used NPI and their results are largely [22]. All eight studies have reported over a 50% prevalence of BPSD (see Table 3). Frequently-reported BPSD include apathy, aggression, agitation and irritability [22,25]. The vast majority of studies (90%) using the NPI to assess BPSD have reported these symptoms as highly prevalent. The studies discussed (see Table 3) indicate the clear consistency of results gained from using the NPI. An additional strength of the NPI is that it is explicitly for the use of investigating the behavioural and psychological domains is common in dementia. This rules out other symptoms patients maybe experiencing which are not related to dementia.

Based on theoretical considerations, the NPI has potential to be a valid tool used to assess BPSD, It is explicitly developed for the use of assessing neuropsychiatric symptoms of dementia. The studies discussed aid in supporting the use of the NPI for future research. It can be assumed that the NPI can be used efficiently to assess the prevalence of BPSD in individuals with learning disabilities.

**Table 3:** Studies using the Neuropsychiatric inventory (NPI) to assess BPSD.

Author, Year, Country	Aim	Measure Used to Assess Behavioural and Psychological Symptoms of Dementia (BPSD)	Participants (*n*)	Main Findings
Lyketsos *et al.* [39], USA	To estimate the prevalence of neuropsychiatric symptoms in dementia	NPI	3608	80% had at least one symptom. The most frequent disturbances were apathy (36%), depression (32%), and agitation/aggression (30%)
Ikeda *et al.* [40], Japan	Investigate the prevalence of mental behavioural disturbances associated with dementia in elderly people	NPI	60	88.3% had shown one or more mental and behavioural disturbances. Common symptoms include Apathy (56.7%), agitation/aggression (35%), aberrant motor behaviour (31.7%), and irritability (31.7%)
Margallo-Lana *et al.* [25], UK	To determine the prevalence of BPSD in care environments, their relationship with severity of dementia and the pattern of psychotropic medication	NPI	231	79% of participants had clinically significant BPSD. Most common symptom was agitation (48%)
Ballard *et al.* [26], UK	Examining the prevalence, incidence, and outcome of the 3 main BPSD (agitation, depression, and psychosis) in care facilities	NPI	136	Prevalence of BPSD was 76% at baseline and 82% at follow-up. Most common symptom was agitation, occurring in 55% of ps
Habio *et al.* [28], China	Investigate prevalence of BPSD in population based community-living persons with dementia	NPI	1271	50.1% had BPSD. The most common symptoms were sleep disturbance (21.9%), irritability (19.6%), apathy (15.7%)
Ballard *et al.* [26], UK	Investigated the impact of BPSD on quality of life in people with dementia	NPI	209	54% had at least one BPSD. Most common symptom was agitation (64%) followed by irritability (45%)
Haung, *et al.* [41], Taiwan	Investigated impact of BPSD on carer burden	NPI	88	84.1% had at least one BPSD. Most common symptom was dysphoria/depression (40.9%), followed by anxiety 37.5%
Neil and Bowie [42], UK	Investigated impact of BPSD on carer burden	NPI	30	BPSD present in 96.2% of ps, most common included hallucinations (62%) and agitation (51%)

### Pilot Study

A pilot study was conducted to examine the prevalence of BPSD in the learning disability population using the neuropsychiatric inventory (NPI-Q). Individuals with learning, disabilities both with and without dementia, were recruited via the Leicestershire Frith Hospital. The participant sample contained six cases with learning disabilities dementia. The remaining three participants did not have a dementia diagnosis, therefore were controls. The results from the pilot study clearly indicated all 12 BPSD were reported more frequently in participants with dementia than without dementia. The most frequent BPSD was night-time behaviours, reported in 66.7% of participants with dementia. The least common BPSD was depression/dysphoria and eating behaviours, which was only present in 16.7% of participants with dementia. Findings suggest a strong prevalence of BPSD in individuals with learning disability and dementia. However, further research is required to be able gain a greater understanding of BPSD.

## 6. Discussion

The aim of this literature review was to describe BPSD prevalence in the general and learning disability population. It has been estimated that BPSD effects up to 90% of all dementia subjects over the progression of their illness [3]. Throughout the majority of dementia diagnostics criteria BPSD have not been included as core features of defining dementia. However, two thirds of people with dementia experience BPSD at any one point during the course of dementia. Investigations of the prevalence of BPSD have produced varied findings across differing settings. The studies used in this literature review have indicated that there is a high prevalence of BPSD in the general population.

It is apparent that there is a gap within research exploring the prevalence of BPSD in individuals with learning disabilities. Findings from upcoming research could lead to a greater understanding of BPSD, thus aid in possibly creating interventions to make symptoms more manageable. The results indicate that individuals with learning disabilities and dementia may also suffer from various psychiatric and behavioural disturbances. However, the differing measures used these studies have made methodological comparisons extremely difficult.

The results suggest that the NPI is a valid measurement used to assess BPSD. The NPI covers 12 major domains of BPSD and is easy to administer. Furthermore, the NPI has been adapted to be administered in a variety of settings such as institutional settings and has been translated into various languages, which potentially makes it a universal tool used for assessing BPSD.

The NPI could be a potentially reliable measure of assessing the prevalence of BPSD in the learning disability population. It is administered to the carer and is dementia focused, which allows it to rule out symptomology from other conditions.

## 7. Conclusions

The overall findings from this literature review imply that BPSD is a highly prevalent issue within dementia. BPSD have been found in those with learning disabilities and dementia, however additional research with valid measures is required to support these findings. The literature review has indicated that further research within this specific area is necessary, especially comparing studies, which use the same tools to assess BPSD. Although the present literature review is not exhaustive due to the lack of research, it highlights the need for future studies to investigate the prevalence of BPSD within individuals with intellectual or learning disabilities.

## References

[1] Gustafson L. (1996). What is dementia?. Acta Neurol. Scand..

[2] Alzheimers Association, What is Dementia. http://www.alzheimers.org.uk/site/scripsdocuments_info.php?documentID=106.

[3] Alzheimer’s Society (2014). Dementia UK.

[4] Knapp M.P., Guerchet M., Comas-Herrera P., Wittenberg A., Adelaja R., King H.B., Salimkumar D. (2014). Dementia UK: Update.

[5] Cooper S.A., Melville C., Jillian M. (2004). People with intellectual disabilities: Their health needs differ and need to be recognised and met. BMJ.

[6] Hammill D.D., Leigh J.E., McNutt G., Larsen S. (1998). A new definition of learning diabilities. Learn. Disabil. Q..

[7] Cooper S.A. (1997). High prevalence of Dementia among people with learning disabilities not attributable to Down’s syndrome. Psychol. Med..

[8] Finnamore T., Lord S. (2007). The use of Dementia Care Mapping in people with a learning disability and dementia. J. Intell. Disabil..

[9] Bush A., Beail N. (2004). Risk Factors for dementia in people with Down’s syndrome: Issues in assessment and diagnosis. Am. J. Ment. Retard..

[10] Rowe J., Lavender A., Turk V. (2006). Cognitive executive function in Down’s syndrome. Br. J. Clin. Psychol..

[11] Zigman W.B., Schupf N., Sersen E., Silverman W. (1996). Prevalence of dementia in adults with and without Down’s syndrome. Am. J. Ment. Retard..

[12] Watchman K. Learning Disabilities and Dementia. http://www.alzheimers.org.uk/site/scripts/documents_info.php?documentID=103.

[13] Van der Linde R.M., Dening T., Matthews F.E., Brayne C. (2013). Grouping of behavioural and psychological symptoms of dementia. Int. J. Geriatr. Psychiatry.

[14] Purandare N., Allen N.H.P., Burns A. (2000). Behavioural and psychological symptoms of dementia. J. Clin. Gerontol..

[15] O’Brien J. (2003). Behavioral symptoms in vascular cognitive impairment and vascular dementia. Int. Psychogeriatr..

[16] O’Donnell B., Drachman D., Barnes H., Peterson H., Swearer J., Lew R. (1992). Incontinence and troublesome behaviors predict institutionalization in dementia. J. Geriatr.Psychiatry.

[17] Rosdinom R., Zarina M.Z., Zanariah M.S., Marhani M., Suzaily W. (2013). Behavioural and psychological symptoms of dementia, cognitive impairment and caregiver burden in patients with dementia. Prev. Med..

[18] Biacnchetti A., Ranieri P., Margiotta A., Trabucchi M. (2006). Pharmacological treatment of Alzheimer’s disease. Aging Clin. Exp. Res..

[19] Stall J., Sacks A., Matheis R., Collier L., Calia T., Hanif H., Kofman E.S. (2007). The effects of Snoezelen (multi-sensory behavior therapy) and psychiatric care on agitation, apathy, and activities of daily living in dementia patients on a short term geriatric psychiatric inpatient unit. Int. J. Psychiatry Med..

[20] McKeith I., Cummings J. (2005). Behavioural changes and psychological symptoms in dementia disorders. Lancet Neurol..

[21] Finkel S.I., Costa e Silva J., Cohen G., Miller S., Sartoorius N. (1996). Behavioral and psychological signs and symptoms of dementia: A consensus statement on current knowledge and implications forresearch and treatment. Int. Psychogeriatric..

[22] Savva M.G., Zaccai J., Matthews F.E., Davidson J.E., McKeith I., Brayne C. (2009). Prevalence, correlates and course of behavioural and psychological symptoms of dementia in the population. Br. J. Psychiatry.

[23] Haupt M., Kurz A., Jänner M. (2000). A 2-year follow-up of behavioural and psychological symptoms in Alzheimer’s disease. Dement. Geriatr. Cognit. Disord..

[24] Chiu M.J., Chen T.F., Yip P.K., Hua M.S., Tang L.Y. (2006). Behavioural and psychologic symptoms in different types of dementia. J. Formos. Med. Assoc..

[25] Margallo-Lana M., Swann A., O’Brien J., Fairbairn A., Reichelt K., Potkins D., Mynt P., Ballard C. (2001). Prevalence and pharmacological management of behavioural and psychological symptoms amongst dementia sufferers living in care environments. Int. J. Geriatric. Psychiatry.

[26] Ballard C., O’Brien J., James I., Mynt P., Lana M., Potkins D., Reichelt K., Lee L., Swann A., Fossey J. (2001). Quality of life for people with dementia living in residential and nursing home care: The impact of performance on activities of daily living, behavioral and psychological symptoms, language skills, and psychotropic drugs. Int. Psychogeriatric..

[27] Brodaty H., Draper B., Saab D., Low L.F., Richards V., Paton H., Lie D. (2001). Psychosis, depression and behavioural disturbances in Sydney nursing home residents: Prevalence and predictors. Int. J. Geriatric. Psychiatry.

[28] Haibo X., Shifu X., Pin N.T., Chao C., Guorong M., Xuejue L., Shiming B., Wenli F., Jun L., Mingyuan Z. (2013). Prevalence and severity of behavioral and psychological symptoms of dementia (BPSD) in community dwelling Chinese: Findings from the Shanghai three districts study. Aging Ment. Health.

[29] Keene J., Hope T., Fairburn C.G., Jacoby R., Gedling K., Ware C.J. (1999). Natural history of aggressive behaviour in dementia. Int. J. Geriatr. Psychiatry.

[30] Duggan L., Lewis M., Morgan J. (1996). Behavioural changes in people with learning disability and dementia: A descriptive study. J. Intell. Disabil. Res..

[31] Cooper S.A., Prasher V.P. (1998). Maladaptive behaviours and symptoms of dementia in adults with Down’s syndrome compared with adults with intellectual disability of other aetiologies. J. Intell. Disabil. Res..

[32] Ravona-Springer R., Davidson M. (2009). Depression and apathy in dementia. Romanian J. Psychopharmacol..

[33] Burt D.D., Loveland K.A., Kay R.L. (1992). Depression and the onset of dementia in adults with mental retardation. Am. J. Ment. Retard..

[34] Prasher V.P., Filer A. (1995). Behavioural disturbance in people with Down’s syndrome and dementia. J. Intellect. Disabil. Res..

[35] Prasher V.P. (1995). Age-specific prevalence, thyroid dysfunction and depressive symptomatology in adults with down syndrome and dementia. Int. J. Geriatr. Psychiatry.

[36] Moss S., Patel P. (1997). Dementia in older people with intellectual disability: Symptoms of physical and mental illness, and levels of adaptive behaviour. J. Intell. Disabil. Res..

[37] Moss S., Patel P. (1995). Psychiatric symptoms associated with dementia in older people with learning disability. Br. J. Psychiatry.

[38] Cummings J.L., Mega M., Gray K., Rosenberg-Thompson S., Carusi D.A., Gornbein J. (1994). The neuropsychiatric inventory comprehensive: Asssessmet of psychopathology in dementia. Neurology.

[39] Lyketsos C.G., Oscar Lopez B.J., Annette L.F., John B., Steven D. (2002). Prevalence of neuropsychiatric symptoms in dementia and mild cognitive impairment: Results from the cardiovascular health study. JAMA.

[40] Ikeda M., Fukuhara R., Shigenobu K., Hokoishi K., Maki N., Nebu A., Komori K., Tanabe H. (2004). Dementia associated mental and behavioural disturbances in elderly people in the community: Findings from the first Nakayama study. J. Neurol. Neurosurg. Psychiatry.

[41] Huang Y.-J., Chieh-Hsin L., Hsien-Yuan L., Guochuan E.T. (2012). NMDA neurotransmission dysfunction in behavioral and psychological symptoms of Alzheimer’s disease. Curr. Neuropharmacol..

[42] Neil W., Peter B. (2008). Carer burden in dementia—Assesing the impact of behavioural and psychological symptoms via self-report questionnaire. Int. J. Geriatr. Psychiatry.

